# Novel track field test to determine V_peak_, relationship with treadmill test and 10-km running performance in trained endurance runners

**DOI:** 10.1371/journal.pone.0260338

**Published:** 2022-01-27

**Authors:** Francisco de A. Manoel, Cecilia S. Peserico, Fabiana A. Machado

**Affiliations:** 1 Department of Physical Education, Cesumar University, Maringá, Paraná, Brazil; 2 Department of Physical Education, Federal University of Lavras, Lavras, Minas Gerais, Brazil; 3 Department of Physical Education, State University of Maringá, Maringá, Paraná, Brazil; 4 Associate Post-Graduate Program in Physical Education UEM/UEL, State University of Maringá, Maringá, Paraná, Brazil; 5 Department of Physiological Sciences, Post-Graduate Program of Physiological Sciences, State University of Maringá, Maringá, Paraná, Brazil; University of Rome, ITALY

## Abstract

**Objectives:**

The aim of this study was to determine the peak running velocity on the track field (V_peak_TF_) based on the laboratory treadmill test (V_peak_T_), and relate the V_peak_ values as well as their correlation with the 10-km running performance in trained endurance runners.

**Method:**

Twenty male trained endurance runners (age: 29.5 ± 5.3 years; V̇O_2max_: 67.5±17.6 ml · kg^-1^·min^-1^) performed three maximum incremental tests to determine the V_peak_: one for V_peak_T_ determination and two to obtain V_peak_TF_ on the official track field (400 m), and a 10-km running performance. During the incremental tests, maximum heart rate (HR_max_), maximal rating of perceived exertion (RPE_max_), and peak lactate concentration (LA_peak_) were determined.

**Results:**

The results showed significant difference between the V_peak_TF_ and V_peak_T_ (18.1 ± 1.2 *vs*. 19.2 ± 1.5 km·h^-1^, respectively), as well as the total time of the tests, the distance traveled and the RPE_max_ determined during the tests. A high correlation was observed between the V_peak_ values (r = 0.94), and between V_peak_TF_ and V_peak_T_ with 10-km running performance (r = -0.95 *vs*. r = -0.89, respectively).

**Conclusions:**

The good agreement and association with V_peak_T_ and high correlation with 10-km running performance demonstrate that the novel track field test is efficient for V_peak_TF_ determination.

## Introduction

The assessment of aerobic variables for the prescription of endurance running is important when considering the training process [1, 2], to determine possible adaptations and prescriptions of training intensities. Although these variables are generally determined in laboratories under controlled conditions [3, 4], the applicability of the results in daily practice conditions is still questionable [5]. Therefore, it is more practical and ecological to determine the variables in an environment directly related to training practice using track field tests [5, 6].

The peak running velocity (V_peak_) is considered an indicator of aerobic fitness, is highly reproducible when determined on treadmill [7], has a high correlation with endurance running performance [8–10] and is sensitive to the training effects [11–13].

Despite the effectiveness of this test in determining Peak running velocity on the laboratory treadmill test V_peak___T_, it is usually performed under laboratory conditions, which tend to relatively deviate from the reality of training and competition for runners [5, 8, 9]. Small differences in the V_peak_ values could impair the entire training program and underestimate or overestimate the required exercise intensity [14–16]. However, some studies have not compared V_peak___T_ with the V_peak_ obtained on the track field (V_peak___TF_) test based on this well-established laboratory treadmill test [8, 9].

Previous studies have compared variables commonly used for endurance training prescription and monitoring (*i*.*e*., maximum aerobic speed—MAS, V_peak_) which were determined during maximum incremental running tests performed on the treadmill and track field [6, 17, 18]. However, it should be noted that the studies mentioned above used different designs. Thus, the determination of the V_peak_TF_, as well as its relationship with V_peak_T_ and endurance running performance, has not yet been determined using a well-established laboratory treadmill test. To the best of our knowledge, this is the first study that determined the V_peak_ in trained endurance runners on the track field using the same protocol established for the treadmill in the design proposed by Machado et al. [9]. The result of the study will be important for coaches and athletes, because with the determination of this variable it is possible to prescribe training sessions both continuous and interval for endurance runners.

Therefore, the aim of this study was to determine the peak running velocity on the track field (V_peak_TF_) based on the laboratory treadmill test (V_peak_T_), and relate the V_peak_ values as well as their correlation with the 10-km running performance in trained endurance runners. We hypothesized that although the V_peak_TF_ is different from V_peak_T_, they have a good relationship; additionally, V_peak_TF_ have a higher correlation to the 10-km running performance in trained endurance runners.

## Methods

### Participants

Twenty male trained endurance runners [mean ± SD (age: 29.5 ± 5.3 years, weight: 61.1 ± 6.9 kg, height: 174.6 ± 4.9 cm, V̇O_2max_: 63.7 ± 14.5 ml·kg^-1^·min^-1^)] with 10-km time running performance of 35.2 ± 1.4 minutes, and mean velocity (MV) of 17.1 ± 0.9 km∙h^-1^ (which represented ≅ 74.6% of the MV of the World record, respectively) took place on this study. All participants were experience in competitive long-distance races with training frequency of 6 ± 1 days∙wk^-1^, and distance of 96.4 ± 23.4 km∙wk^-1^, who presented medical clearance to perform exhaustive physical tests. The article adheres to the ethical standards in sports and exercise science research, with the consent of the participant informed in writing to carry out the study and anonymity of its data [19]. The experimental protocol was approved by the University’s Human Research Ethics Committee (#1.889.751/2017) and all participants learned information about a methodology of work, as well as risks and collateral.

### Study design

After being familiarized with the rating of perceived exertion (RPE) scale and the equipment to be used in the evaluations (*e*.*g*., motorized treadmill), the participants performed one maximum incremental test on the treadmill under laboratory conditions (temperature = 21 ± 1°C and relative humidity = 55–60%) and two maximum incremental tests on the official track field for V_peak_ determination. The first test was carried out to adapt the participants to the track field test, and the second one was used to determine the V_peak_TF_. In addition, 10-km running performance was performed on the official track field. The tests were performed at the same time of the day (between 5:00 and 9:00 p.m.) under similar climatic conditions (temperature = 25–29°C and relative humidity = 50–60%) and separated by 1-week interval. The total time, heart rate (HR), RPE and lactate concentrations ([La]) were monitored during all tests.

### Determination of V_peak_ on the treadmill (V_peak_T_)

To determine the V_peak_T_, a continuous and incremental test was used with velocity increments of 1 km·h^−1^ every 3-min without breaks between stages. The V_peak_T_ was assessed on a motorized treadmill (Super ATL; Inbrasport^®^, Porto Alegre, Brazil) with a gradient set at 1% [20]. After 3-min warm-up walking at 6 km·h^−1^, the test started with an initial velocity of 8 km·h^−1^, followed by an increase of 1 km·h^−1^ every 3-min until volitional exhaustion (*i*.*e*., participant was unable to continue running) [9], and when at least two of the following criteria were met: (1) peak lactate concentration (LA_peak_) ≥ 8 mmol L^−1^, (2) maximum HR (HR_max_) ≥ 100% of endurance-trained age-predicted HR_max_ using the age-based “206–0.7 × age” equation [21] and (3) maximum RPE (RPE_max_) ≥ 18 in the 6–20 Borg scale. If the last stage was not completed, the V_peak_T_ was calculated based on the partial time completed in the last stage achieved from the equation proposed by Kuipers et al. [22]: V_peak_T_ = V_complete_ + (Inc × t/T), in which V_complete_ is the running velocity of the last complete stage, Inc is the velocity increment (*i*.*e*., 1 km·h^−1^), t is the number of seconds sustained during the incomplete stage, and T is the number of seconds required to complete a stage (*i*.*e*., 180 s).

HR was monitored during all tests (Polar^®^ RS800sd; Kempele, Finland) and HR_max_ was defined as the highest HR value recorded during the test. RPE was also monitored during all tests by using a 6–20 Borg scale [23], and the highest RPE value was adopted as the RPE_max_. Earlobe capillary blood samples (25 μl) were collected into a capillary tube at the end of the tests (time zero of recovery) and at the third, fifth, and seventh minutes of passive recovery with participants seated in a comfortable chair. The [La] was evaluated only at the end of the test and LA_peak_ was defined for each participant as the highest post-exercise [La] value.

#### Determination of V_peak_ on the track field (V_peak_TF_)

The test used to determine the V_peak_TF_ was the same as the one used for the determination of V_peak_T_. The test was a continuous and incremental test was used with increments of 1 km·h^-1^ every 3-min without breaks between stages. The velocity during the test was controlled by sound signals. Participants should cross the lines marked by cones, which were distributed on the track field every 25 m, with at least one foot simultaneously to the beep [24]. The interval between the beeps at each stage decreased every three minutes, and the higher beep indicate that a new stage was starting. Each three minutes was a time reduction between beeps with the objective to increment the velocity, that is, at each velocity increment, the participants should exceed a greater number of cones (travel a greater distance) in the interval of 3-min compared to the previous velocity (Table 1). The test was finished by voluntary exhaustion of the participant or when the evaluator identified that the participant failed to cross the reference lines with one of two feet for two consecutive times [24].

**Table 1:** Test characteristics for determination of V_peak_ on the track field (V_peak_TF_).

Stages (km·h^-1^)	Number of cones traversed per stage	Interval between beeps (s)	Stages (km·h^-1^)	Number of cones traversed per stage	Interval between beeps (s)
**6.0**	12	15.0	**14.0**	28	6.4
**8.0**	16	11.3	**15.0**	30	6.0
**9.0**	18	10.0	**16.0**	32	5.6
**10.0**	20	9.0	**17.0**	34	5.3
**11.0**	22	8.2	**18.0**	36	5.0
**12.0**	24	7.5	**19.0**	38	4.7
**13.0**	26	6.9	**20.0**	40	4.5

The tests were performed at the same time of the day (between 5:00 and 9:00 p.m.) under similar climatic conditions (temperature = 25–29°C and relative humidity = 50–60%). During the tests, HR, RPE and [La] were monitored following the same procedures described previously.

### 10-km running performance

Performance was undertaken on the track field preceded by 10-min warm-up. Participants were requested to run as fast as possible and the time was recorded every 400 m. Mineral water was provided *ad libitum* in cups throughout trials, so that participants could hydrate themselves as they were used to do in long-distance races. The 10-km mean velocity (MV) for each trial was calculated by dividing the total distance by the trial duration. Additionally, partial MVs were calculated in three phases: (1) start (first 400 m), (2) middle (400–9600 m) and (3) end (last 400 m), as previously reported [25, 26].

### Statistical analyses

All statistical analyses were performed using the software *Statistical Package for the Social Sciences* (SPSS^®^ v.20, Inc., Chicago, IL, USA). Data normality was verified by the Shapiro-Wilk test. The variables are presented as mean ± standard deviation (SD). The comparisons between the V_peak_TF_ and V_peak_T_ tests were performed by the Student’s paired t-test. To examine the correlation and confidence interval (CI) between both V_peak_ and 10-km running performance, Pearson product-moment correlations were performed. Correlation coefficients (R) were interpreted using the following qualitative descriptors: trivial (< 0.1), small (< 0.3), moderate (0.3–0.5), large (0.5–0.7), very large (0.7–0.9), nearly perfect (> 0.9), and perfect (1.0) [27]. Simple linear regression analyses were used to generate a predictive equation V_peak_TF_ and from V_peak_T_. The Bland-Altman analysis [28] was used to calculate the bias (difference between the means) between the V_peak_TF_ and V_peak_T_ with the respective limits of agreement for a 95% interval (LoA = bias ± 1.96 mean ± SD). Hopkins spreadsheets were used to calculate intraclass correlation coefficient (ICC). Results for MV recorded at the three different points during performances were compared using two-factor ANOVA for repeated measures followed by the LSD *post hoc* test for multiple comparisons. For all analyses a significance level of *P* < 0.05 was adopted.

## Results

There was no significant difference between V_peak_TF_ and V_peak_T_ as well as for the total time of the tests, the distance travelled and RPE_max_ determined during the tests, with higher values obtained on the treadmill test (Table 2). A higher ICC values was found for V_peak_ values (Table 2).

**Table 2:** Comparison, association and agreement between variables obtained during the track field and treadmill tests (N = 20).

Variable	Track	Treadmill	*P*	% Diff	ICC	Bias
	Field			(CI 95%)	(CI 95%)	(95% LoA)
V_peak_ (km·h^-1^)	18.1 ± 1.2	19.2 ± 1.5*	< 0.001	6.10 (1.9–11.4)	0.94 (0.86–0.98)	1.11 (-0.02–2.2)
Duration (min)	36.2 ± 3.4	39.7 ± 4.2*	< 0.001	8.7 (1.2–17.9)	0.92 (0.80–0.97)	3.40 (0.1–6.6)
Distance (km)	7.6 ± 1.1	8.7 ± 1.4*	< 0.001	14.10 (1.1–25.9)	0.93 (0.84–0.97)	1.10 (0.02–2.1)
HR_max_ (bpm)	184.0 ± 10.2	185.0 ± 9.5	0.096	1.00 (-3–3)	0.95 (0.88–0.98)	0.90 (-4.8–6.6)
RPE_max_ (AU)	19.3 ± 1.1	19.9 ± 0.5*	0.012	3.10 (0–11)	0.46 (0.04–0.75)	-0.05 (-0.46–0.4)
LA_peak_ (mmol·L^−1^)	8.2 ± 1.9	9.1 ± 2.9	0.219	15.6 (-26–35)	0.25 (-0.20–0.62)	0.50 (-1.9–2.9)

Note: V_peak_TF_, Peak running velocity on the track field; V_peak_T_, Peak running velocity on the laboratory treadmill test; CI, confidence interval; ICC, intraclass correlation coefficient; LoA limits of agreement (LoA = bias = 1.96 SD).

*P < 0.05 in relation to the track field test.

**Fig 1:**
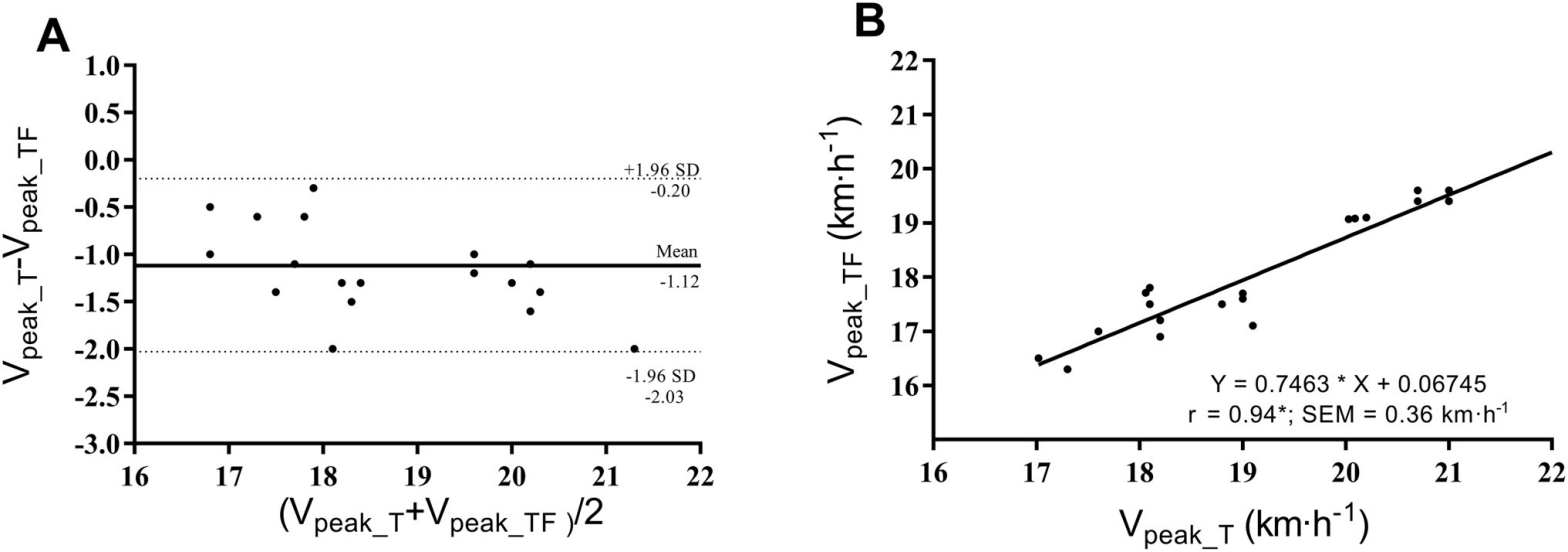
A) Bland-Altman plots indicating the agreement between the V_peak_ values obtained on track field and the treadmill tests. 1B) Linear regression relationship between the V_peak_ values determined on the treadmill running and track field tests. Note: V_peak_TF_ Peak running velocity on the track field; V_peak_T_ Peak running velocity on the laboratory treadmill test. *P < 0.05.

Fig 1A shows the good agreement between the V_peak_ values. Fig 1B demonstrates a significant and high linear correlation between the V_peak_TF_ and V_peak_T_. Assuming a standard error of 0.36 km·h^−1^, the resulting equation was:

$${\text{V}}_{\text{peak}\_\text{TF}}(\text{km}\cdot {\text{h}}^{-1})=0.75\times {\text{V}}_{\text{peak}\_\text{T}}(\text{km}\cdot {\text{h}}^{-1})+0.07$$

**Fig 2:**
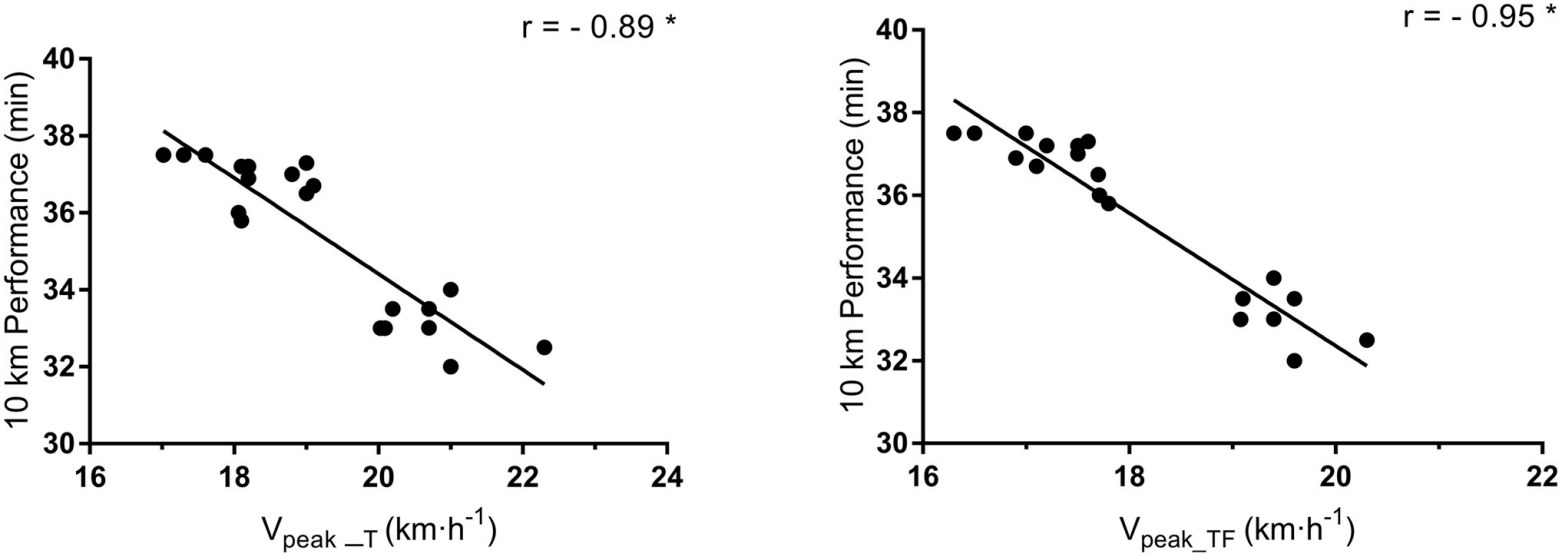
Correlation between both V_peak_ values with 10-km running performance time. Note: V_peak_TF_, Peak running velocity on the track field; V_peak_T_, Peak running velocity on the laboratory treadmill test. *P < 0.05.

Fig 2 shows the association between both V_peak_ and the 10-km running performance. High and significant correlation was found between 10-km running performance time and V_peak_TF_ (r = -0.95; CI = -0.88 to -0.98) and V_peak_T_ (r = -0.89; CI = -0.74 to -0.96), respectively.

**Fig 3:**
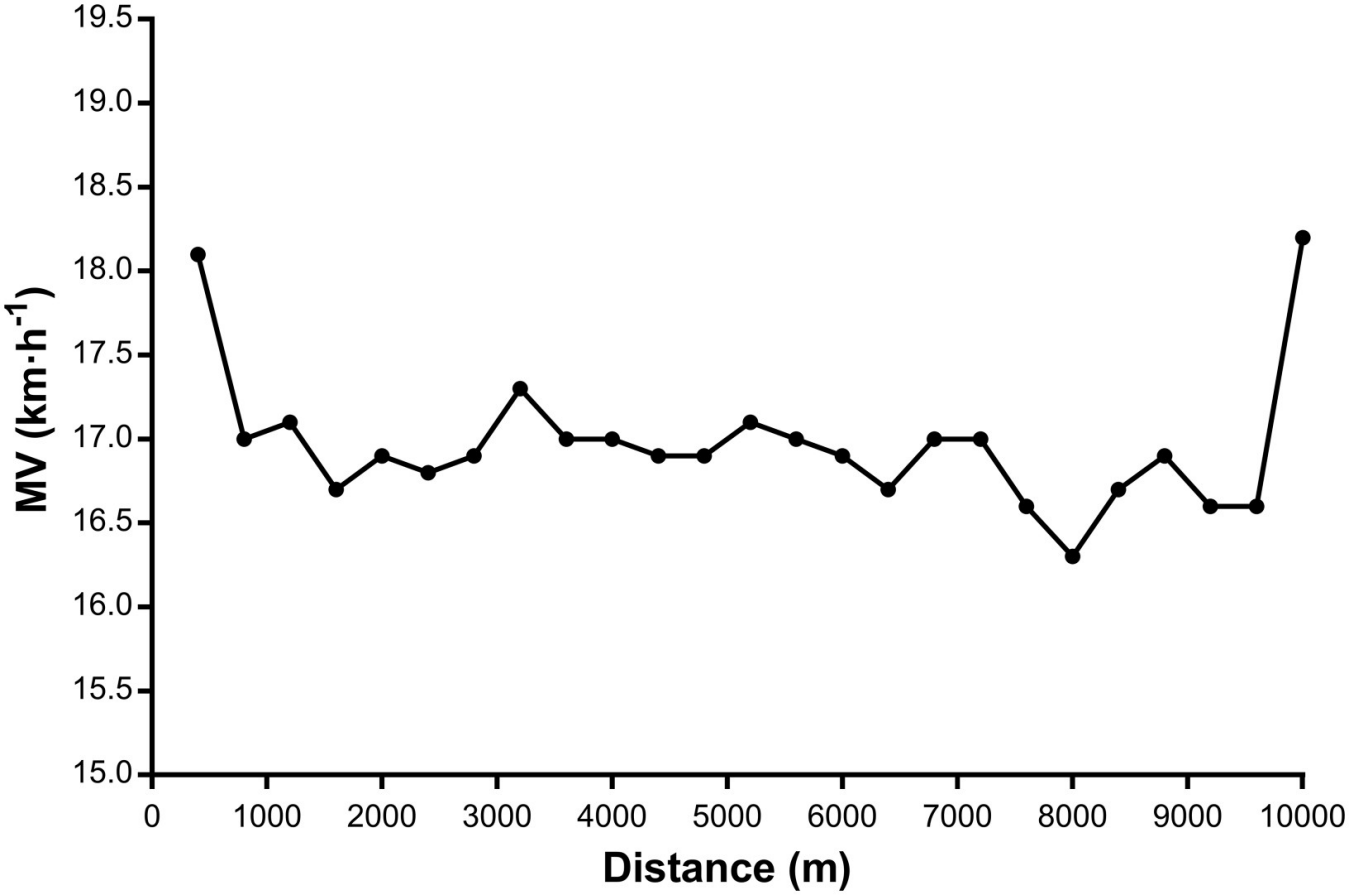
Mean Velocity during the different phases adopted by the participants of the present study for 10-km running performance. Note: MV, Mean Velocity.

Fig 3 shows the variation of MV according to distance, which helped determine that the participants used the “U” running pace as a test strategy in 10-km running performance.

## Discussion

The aim of this study was to determine the peak running velocity on the track field based on the laboratory treadmill test, and relate the V_peak_ values as well as their correlation with the 10-km running performance in trained endurance runners. The main findings were that despite the difference between V_peak_ values both V_peak_ values were associated and have good agreement, and V_peak_TF_ had a higher correlation with the 10-km running performance than V_peak_T_, which confirm the initial hypothesis.

The results showed that V_peak_TF_ was significantly lower compared to V_peak_T_. As demonstrated in the present study, some researchers compared incremental tests performed on the treadmill and track field and observed higher values for the variables (*e*.*g*., MAS and V_peak_) determined on the treadmill [6, 17, 18]. Simillarly, Pallares et al. [6] showed that V_peak_ and MAS obtained on the treadmill test (increments of 1 km·h^-1^ every 1-min) were similar when compared to the values measured in a new short track test (same treadmill protocol) performed in the field. Metsios et al. [18] observed that the MAS determined during the treadmill test (increments of 1 km·h^-1^ every 2-min) was overestimated by 8% when compared with the track field test (increments of 0.5 km·h^-1^ every 1-min), which is very similar to the present investigation (≅ 6%). However, previous studies [6, 17, 18] compared protocols in the treadmill and track field with different designs. According to Kuipers et al. [22], it is important to use the same test design for comparison and validation of a given variable, such as V_peak_, which is directly influenced by the protocol design [8, 9, 21].

Studies point out that there is a great difference between running on the treadmill and running in the field/track [29–31]. Considerable kinematic differences exist, and the mechanisms of the march are involved in the treadmill race different from those of the race on the track [32–34], as well as biomechanical differences (*e*.*g*., when running on a treadmill, the pass frequency is higher compared to the track, while the stride length is higher on the track) [29]. Although the study did not evaluate these factors, we consider that they may have contributed to the final differences found between the tests to determine. Furthermore, we highlight that the environmental conditions, which are variables that are better controlled when the tests are performed on the treadmill, contribute to the differences between V_peak_TF_ and V_peak_T_ (r = -0.95 *) in the present study [35]. However, it is important to emphasize that the data obtained from the track field tests were closer to the competitive reality and training of the runners [36].

Despite the differences between both V_peak_ tests, the ICC and Bland-Altman demonstrated that V_peak_TF_ is highly associated with the well-established V_peak_T_; however, the Bland-Altman demonstrated a bias of 1.1 km·h^-1^ and a percentage difference of 6.1% between the V_peak_ values. In contrast, Pallarés et al. [6] with trained male athletes demonstrated that the V_peak_ determined by the novel short track test had high ICC (0.96), low bias (-0.1) and a % Diff of -0.6% when related to the V_peak_ obtained on the treadmill using the same protocol. This great similarity between the two protocols demonstrated by Pallarés et al. [6] can be related to the fact that the authors used a gas analyzer for the treadmill test. It is important to emphasize that the present study used clean protocols (*i*.*e*., without using gas analyzers in both tests), which contributed to the runners staying longer on the treadmill test and caused the difference between the V_peak_ values.

In relation to other variables determined in the V_peak_ tests, it was observed significant higher values for RPE_max_ when determined on treadmill compared to track field. This can be justified by the fact that runners reach an extra stage during the treadmill test, in addition, runners have a perception of greater velocity on the treadmill due to the need for greater balance and coordination, the increased demand for attention and vision, and the fear of falling [37]. However, no significant difference was observed for the HR_max_ and LA_peak_ values, demonstrating that both incremental protocols attained similar maximal effort responses. The similar result was observed by Pallarés et al. [6] who also found no difference in HR_max_ on comparing incremental tests on the track field and treadmill. It should be noted that these variables were used to identify the physiological responses generated by effort, in addition to being used as a parameter to identify the maximum effort during the incremental test [38, 39].

Another important finding of the present study is that the V_peak_TF_ showed a higher correlation with the 10-km running performance than the V_peak_T_ (r = -0.95 *vs*. -0.89, respectively), demonstrating that improvements in the V_peak_TF_ during a training period can directly reflect performance changes. Previous studies also observed high correlations (between -0.80 and -0.93) between V_peak_T_ and performances ranging from 3 to 90 km [9, 10, 40], however, no study has demonstrated the correlation between V_peak_TF_ and performance. It is suggested that the high correlation of V_peak___TF_ is because the test location (*i*.*e*., outdoor) was similar to that of the performance, and was where the runners usually compete. This result also reinforces the great practical application of V_peak_TF_ as a training prescription variable.

To complete a 10 km performance, participants adopted the "U" strategy [41]. This strategy is commonly used by moderate and high-performance runners [26, 42]. After assessing the contribution of some physiological and muscular variables to the rhythm strategy adopted during the 10 km running performance, Bertuzzi et al. [25] concluded that V_peak_, V̇O_2max_ and 1 maximum repetition are the variables that best explain the performance in the intermediate phase (0.4–9.6 km) and only V_peak_ in the final phase (9.6–10 km), reaffirming its high performance prediction capacity for this type of test.

Despite the important findings, this study had some limitations such the absence of other test using the gas analyzer to obtain ventilatory parameters; however, future studies can investigate the relationship between V_peak_TF_ and ventilatory parameters. Other limitation was the lack of a dietary recall to control and standardize the same diet before the testing sessions; however, it was recommended for the participants to maintain the same diet pattern before each test.

The results of this study have important practical implications for endurance coaches, practitioners, and runners in terms of the prescription of aerobic training loads on the track. This is because of the practicality and ecological validity of the V_peak_TF_ test, which is determined in an environment directly related to the training location of runners. Further, this test is suitable for the simultaneous evaluation of several runners. In order to prescribe endurance training using the variable, it is suggested to use the intensity of 75 ± 4% of V_peak_ for continuous training sessions and intensities of 100% ± 2% of V_peak_ for long interval training session [11–13] and 120% ± 2% of V_peak_ for short interval training session [13].

## Conclusion

In conclusion, the good agreement and association with V_peak_T_ and the high correlation with 10-km running performance demonstrate that the novel track field test is efficient for V_peak_TF_ determination. Future studies should verify the reproducibility of this novel track field test in runners with different levels of performance.

## Supporting information

S1 Data (XLSX)
